# Fast and Interpretable Machine Learning Modeling of Atmospheric Molecular Clusters

**DOI:** 10.1021/acs.jpca.5c06950

**Published:** 2026-01-15

**Authors:** Lauri Seppäläinen, Jakub Kubečka, Jonas Elm, Kai R. Puolamäki

**Affiliations:** † Department of Computer Science, 3835 University of Helsinki, Pietari Kalmin katu 5, 00560 Helsinki, Finland; ‡ Department of Chemistry, 1006 Aarhus University, Langelandsgade 140, 8000 Aarhus C, Denmark

## Abstract

Understanding how atmospheric molecular clusters form and grow is key to resolving one of the biggest uncertainties in climate modeling: the formation of new aerosol particles. While quantum chemistry offers accurate insights into these early-stage clusters, its steep computational costs limit large-scale exploration. In this work, we present a fast, interpretable, and surprisingly powerful alternative: the *k*-nearest neighbor (*k*‑NN) regression model. By leveraging chemically informed distance metrics, including a kernel-induced metric and one learned via metric learning for kernel regression (MLKR), we show that simple *k*-NN models can rival more complex kernel ridge regression (KRR) models in accuracy while reducing computational time by orders of magnitude. We perform this comparison with the well-established Faber–Christensen–Huang–Lilienfeld (FCHL19) molecular descriptor; however, other descriptors (e.g., FCHL18, MBDF, and CM) can be shown to have similar performance. Applied to both simple organic molecules in the QM9 benchmark set and large data sets of atmospheric molecular clusters (sulfuric acid–water and sulfuric–multibase–base systems), our *k*-NN models achieve near-chemical accuracy, scale seamlessly to data sets with over 250,000 entries, and even appears to extrapolate to larger unseen clusters with minimal error (often nearing 1 kcal/mol). With built-in interpretability and straightforward uncertainty estimation, this work positions *k*-NN as a potent tool for accelerating discovery in atmospheric chemistry and beyond.

## Graphical abstract

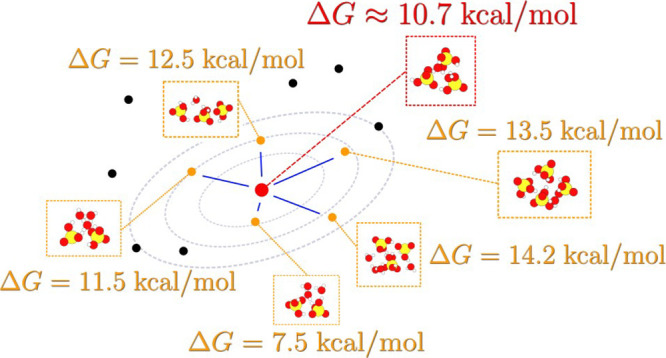

## 1 Introduction

New particle formation (NPF) is the dominant source of aerosol particles in the atmosphere.^1–3^ NPF contributes substantially to the global cloud condensation nuclei (CCN) budget, with 10–80% of the number concentration, depending on region.^4–6^ As specified by the most recent IPCC report,^7^ the largest source of uncertainty in estimating Earth’s current and future radiative balance originates from our lack of understanding of how aerosol particles are formed and how many of them eventually reach CCN sizes of roughly 50–100 nm.

The NPF process is initiated by the formation of strongly bound atmospheric molecular clusters.^8^ Measuring the cluster formation process using current experimental instrumentation is challenging, as soft ionization mass spectrometer techniques are not able to identify all clusters simultaneously.^9^ Accurate quantum chemical (QC) calculations can capture clustering of single molecules up to small 1–2 nm particle sizes. However, the desired accuracy comes at a cost of steep computational scaling with the studied system size. For instance, the density functional theory methods typically employed for obtaining cluster structures scale roughly as $O\left(N^{4}\right)$ , where *N* represents the number of basis functions or orbitals, and highly accurate CCSD­(T) methods scale roughly as $O\left(N^{7}\right)$ . Localization approaches such as DLPNO–CCSD­(T),^10–12^ PNO–CCSD­(T),^13,14^ or LNO–CCSD­(T)^15–18^ can bring the scaling down, but still remain expensive on large cluster systems.

A promising strategy to overcome the computational costs of quantum chemical methods is the application of machine learning (ML). ML methods have been widely applied in chemistry as powerful tools in a broad spectrum of tasks, including drug discovery,^19^ reaction pathway discovery,^20^ estimation of material properties,^21^ and the analysis of complex molecular data sets generated by experimental techniques.^22–24^ Due to their relatively low marginal computational cost, machine learning methods can be trained with large data sets and can hence rival the performance of QC calculations. Furthermore, the inference cost, i.e., applying machine learning methods to novel data points, is often significantly lower than the training cost. Thus, machine learning methods are increasingly utilized for interpolating results from QC calculations, as full replacements for these calculations, as well as prescreening tools to choose the most promising candidate structures for more accurate calculations.^25–28^ However, the computational costs for ML methods cannot be entirely ignored either. These costs can limit the applicability of ML, particularly with large data sets, alongside the lack of mature, user-friendly software.^29^ Also, estimating prediction uncertainties and extrapolation to outside training data are nontrivial problems without off-the-shelf solutions, even with ML.

Due to the lack of appropriate databases, ML has only been scarcely applied to atmospheric chemistry.^30^ In recent years, however, a variety of ML techniques have been explored for predicting key atmospheric properties. For instance, molecular saturation vapor pressures modeled via kernel ridge regression (KRR^31^), extreme minimal learning machine (EMLM^32^), and neural networks (NN^33^) have shown promise. Neural networks, in particular, have become a popular choice across chemistry for their flexibility and ability to model highly nonlinear relationships. Recent work has demonstrated their ability to predict binding energies of atmospheric clusters with impressive accuracy.^34–37^ However, the benefits of NNs often come at the cost of interpretability and computational complexity, especially during training. In many practical scenarios, neural networks require significantly larger data sets to avoid overfitting and can be prone to unpredictable generalization behavior outside the training domain. Additionally, their “black-box” nature may limit chemical insight, an important factor when trying to understand the physical principles driving atmospheric cluster formation.

In contrast, kernel-based models such as KRR offer a compelling balance between accuracy and transparency. Kubečka et al. introduced KRR combined with Δ-learning^38^ to predict cluster binding energies of acid–base clusters with sub-1 kcal/mol accuracy.^39^ In addition, Knattrup et al. demonstrated that KRR can even be used to extrapolate beyond the training database regarding system size.^40^ However, as the reference database increases in size beyond 10^4^ data points, both training and inference times become slow, making KRR less practical for large data sets.

The success of KRR in modeling chemical systems implies that other instance- or similarity-based models may also perform well. Perhaps the simplest instance-based model is the well-studied *k*-nearest neighbors (*k*‑NN) regression model, where a prediction for a novel item is formed as a (weighted) average of the labels of the *k* nearest data points in the training set.^41^ Using tree-based data structures, the construction of the data structure (“training” the *k*‑NN model) will take $O\left(pn{\left(\log n\right)}^{2}\right)$ time and the cost of inference (“prediction”) can be made to scale as O(klogn) , where *n* is the number of training data points, *k* is the number of neighbors, and *p* is the dimensionality of the data vectors (e.g., cluster descriptors).^42^ *k*-NN is therefore expected to incur much lower computational costs than KRR as the data set size increases. Besides increased speed, a *k*‑NN model is also readily interpretable; the user can inspect the nearest neighbors (or their 3D structure) to understand how the model produces predictions. The primary challenge in employing a *k*‑NN model is determining an effective distance metric in high-dimensional space. Without careful consideration for the choice of metric, the notion of “nearest neighbors” can become meaningless with as few as 10–15 features.^43^

In this paper, we address this challenge by employing metric learning or, as an alternative option, deriving a distance measure from a kernel function that has been shown to perform well in conjunction with a KRR model. Our main contributions are to describe and evaluate several *k*‑nearest neighbor modeling strategies for predicting the properties of chemical systems. We demonstrate that our methods yield fast predictions for the chemical properties of acid–base clusters, with a computational cost orders of magnitude smaller than conventional machine learning methods, at only a slight cost in prediction accuracy. Furthermore, our proposed method is interpretable and readily allows for uncertainty estimation.

## 2 Experimental and Theoretical Methods

### 2.1 Theory

In this section, we first define the machine learning problem we aim to solve in concrete terms. We then describe KRR and *k*-NN models and the specific algorithms we use to mitigate the curse of dimensionality in *k*‑NN. Finally, we briefly introduce the FCHL descriptor we use to convert 3D structures of chemical systems to training data suitable for the machine learning models.

#### 2.1.1 Problem Definition

Let $D=\{(x_{1},y_{1}),\cdot \cdot \cdot ,(x_{n},y_{n})\}$ be a data set consisting of data points **x**_*i*_ and labels *y*_*i*_, where *i* ∈ {1, ···, *n*}. The data points **x**_*i*_ can be, e.g., molecular descriptors (see examples in Section 2.1.4). The labels correspond to some chemical property of interest, such as the electronic binding energy. In machine learning, we aim to find the function *f*(**x**) which minimizes the value of a chosen loss function $L(y_{i}^{\prime },f(x_{i}^{\prime }))$ . In this paper, we use the mean absolute error (MAE) as the loss function for evaluation:

$$L(y_{i}^{\prime },f(x_{i}^{\prime }))=\frac{1}{n}\sum_{i=1}^{n}|y_{i}^{\prime }-f(x_{i}^{\prime })|$$ 1

Unless otherwise specified, we present the losses on a separate test (validation) set, which is not used in training.

#### 2.1.2 Instance-Based Models

Machine learning methods can be divided into two categories: parametric and nonparametric models.^44^ In parametric models, the aim is to learn a set of parameters θ for a fixed-form function *f* by minimizing the loss on a data set D . Examples include ordinary least-squares linear regression and the more complex, and increasingly ubiquitous, neural networks. Nonparametric models, on the other hand, assume no fixed form for *f*. In this paper, we focus on instance-based nonparametric models, which learn by “memorizing” the training data and produce predictions by leveraging some similarity metric between the training data and novel data points. Such instance-based models can be expressed as

$$f(x^{\prime })=\sum_{j\in J}w(x^{\prime },x_{j})y_{j}$$ 2

where *w*(**x**′, **x**_*j*_) is a weighting function between the novel item **x**′ and data points **x**_*j*_ in a (sub)­set J⊆D of the training data. Examples of the weighting function include similarity- or distance-based functions, such as kernel functions.

In computational chemistry, one of the commonly used instance-based models is kernel ridge regression (KRR).^45^ As the name suggests, KRR builds on ridge regression, a classic statistical model that incorporates a quadratic penalty into the linear regression parameters to mitigate overfitting. KRR increases the model flexibility by first casting the input data points **x** to a feature space, denoted by ϕ­(**x**). Usually, ϕ is a nonlinear function and ϕ­(**x**) has higher dimensionality than the original input space. Due to the “kernel trick,” the data points do not explicitly need to be projected into the feature space; merely defining the dot product between data points in this space suffices. The dot product function *k*(**x**, **x**′) = ϕ­(**x**) · ϕ­(**x**′) is referred to as the kernel, and it measures the similarity between data points in the feature space. Now the prediction from a KRR model can be expressed as a weighted sum of the training labels:

$$f_{\mathrm{KRR}}(x)=\sum_{i=1}^{n}\alpha_{i}k(x_{i},x)$$ 3

from which the optimal weights can be solved from the following matrix equation:

$$\alpha =(K+\lambda I_{n}{)}^{-1}y$$ 4

Here, the kernel matrix **K** consists of kernel evaluations between training data points: **K**_*i*,*j*_ = *k*(**x**_*i*_, **x**_*j*_), λ is the ridge regularisation penalty and **I**_*n*_ is a *n* × *n* identity matrix. In the terms of eq 2, the set *J* is the full training data set and the weighting function is *w*(**x** *′*, **x**_*j*_) = (**K** + λ**I**_*n*_)^−1^ *k*(**x** *′*, **x**_*j*_). Computationally, finding **α** by solving eq 4 is expensive as it involves inverting the *n* × *n* kernel matrix **K** + λ**I**_*n*_, which is an $O\left(n^{3}\right)$ operation in practice,^46^ even though $O\left(n^{2.376}\right)$ matrix inversion algorithms exist.^47^ Furthermore, while training the model incurs this cost only once, inference for *m* samples is O(mn) , which can become prohibitively expensive for large *n*, especially if evaluating the kernel function *k* is nontrivial.

Properties of chemical systems can be divided into extensive properties, which are size- or scale-dependent, and *intensive* properties, which are size-independent. In this paper, we focus on electronic binding energies, which are an extensive property; larger systems generally have lower (more negative) electronic binding energies. One approach to model extensive properties with KRR is to assume that the extensive property can be decomposed into a sum of atomic contributions. The formulation of KRR in this case stays the same, save for rewriting the kernel as a sum of pairwise atomic kernels.^48^

Accuracy of ML models can be further enhanced by using Δ‑learning, a hybrid approach combining machine learning with fast quantum chemical calculations.^49^ In Δ‑learning the aim is not to directly predict a property, such as electronic binding energy, but instead a correction term between, for example, a related quantity (often more easily computed) and the target property (such as between energy and enthalpy), between different geometries (such as isomers of the same compound), or between two quantum chemical properties obtained at different levels of theory. In a previous work, Kubečka et al. have shown how a Δ‑learning approach with KRR can achieve results within chemical accuracy when applied to atmospheric cluster data.^39^ Δ‑learning is not limited to KRR and can be applied in a wide range of ML methods. Additionally, the approach can also be used to optimize computational cost. When the correction term (Δ) represents the difference between two quantum chemical methods, or between a target property and a related, more easily computed quantity, Δ‑learning allows predictions from a simpler or less accurate model to be adjusted toward those of a more accurate, higher-level method. This can significantly improve accuracy at a marginal computational cost increase.

In contrast to a KRR model, in a *k*-NN model, each prediction for a novel item is simply the (weighted or unweighted) average of the labels of the *k* nearest neighbors of that item in the training set:

$$f_{\mathrm{k‐NN}}(x)=\frac{1}{\underset{i\in N_{k}(x,d)}{\sum }w_{i}(x)}\underset{i\in N_{k}(x,d)}{\sum }w_{i}(x)y_{i}$$ 5

where $N_{k}(x,d)$ denotes the set of *k* nearest neighbors for the item ***x*** as per the distance measure *d*(**x**, **x**_*i*_). In the unweighted variant, the weight vector is a unit vector (**w** = **1**) and the prediction is the mean of the nearest labels. However, if the labels can be assumed to vary smoothly with increasing distance, the accuracy of the predictions can be increased by weighing the labels with the reciprocal of the distance: *w*_*i*_ = 1/*d*(**x**, **x**_*i*_). These two are the standard choices for weighting and are hence what we study in this paper. The simplest *k*‑NN training consists of simply memorizing the training samples and hence has an O(np) complexity, where *p* is the dimensionality of the data vectors. Inference for a novel item is O(knp) for a naïve implementation. However, with a tree-based data structure, inference complexity can be pushed down to O(klogn) with slightly higher training cost of $O\left(pn{\left(\log n\right)}^{2}\right)$ needed for building the tree data structure used to find the nearest neighbors efficiently.^42^

#### 2.1.3 *k*-NN Algorithms

The main issue limiting the applicability of nearest neighbor models is the curse of dimensionality. As the dimensionality of the input space increases, the distinction between the Euclidean distances of data points becomes less and less pronounced; the higher the dimensionality, the more equal the distances typically become.^43^ If a large subset of data points is nearly the same distance away from the query item, it is difficult to meaningfully distinguish the nearest neighbors. Hence, the key to applying *k*‑NN to high-dimensional data, such as molecular descriptors, is a well-specified distance metric. We employed two approaches to finding a suitable distance metric for comparison: kernel-induced distance and metric learning. The two approaches are depicted in the *k*‑NN modeling pipeline in Figure 1.

**Figure 1:**
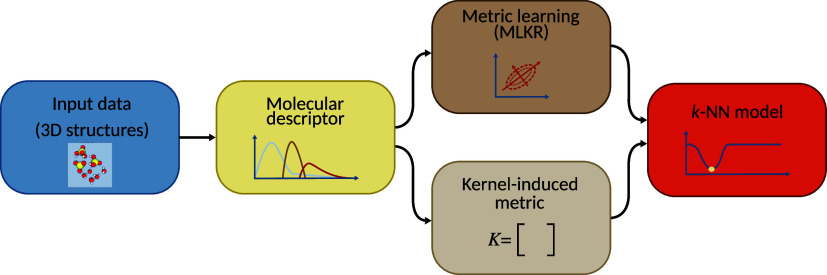
Schematic of the *k*‑NN pipeline. The raw data consist of atomic positions in 3D space, from which we derive molecular descriptors (representations). Then, using one of two different distance measures, a learned one based on the MLKR algorithm and a metric derived from the KRR kernel, we construct the final *k*-NN model.

KRR with the Faber–Christensen–Huang–Lilienfeld (FCHL) representation has shown promising results in the past,^39^ and the key property for this performance is the use of a kernel function *k* that can meaningfully measure the similarity between chemical systems. If we have access to a good similarity metric, a natural assumption is that we can construct a distance metric from it. This is indeed the case, provided certain assumptions are made about the properties of the kernel. The kernel-induced metric^50^ is defined as

$$d(x_{i},x_{j})=\sqrt{k(x_{i},x_{i})+k(x_{j},x_{j})-2k(x_{i},x_{j})}$$ 6

i.e., as the difference between self-similarities and cross-similarity of data points *i* and *j*. This definition is just a reformulation of the distance between the data points in the high-dimensional space:

$$\left|\right|\phi_{i}-\phi_{j}|{|}^{2}=\left(\phi_{i}-\phi_{j}\right)\cdot \left(\phi_{i}-\phi_{j}\right)=\phi_{i}\cdot \phi_{i}+\phi_{j}\cdot \phi_{j}-2\phi_{i}\cdot \phi_{j}$$ 7

written by using definition of the kernel as the dot product in the high-dimensional space ϕ­(**x**) of data point **x**, i.e., *k*(**x**, **x** *′*) = ϕ­(**x**) · ϕ­(**x** *′*). The distance function *d* is a valid pseudometric if *k* is a positive definite kernel, which is usually assumed and is the case for the kernels used in this manuscript. The FCHL kernel is a sum of pairwise atomic radial basis function (RBF) kernels,^51^ which are positive definite, and it can be trivially shown that a sum of positive definite matrices is also a positive definite matrix. Hence, when using the FCHL kernel, the induced kernel distance *d* is a valid distance metric and can be used to create a *k*‑NN model. However, when using KRR to model an extensive property with a kernel composed of a sum of pairwise atomic kernels, the kernel is not normalized, meaning that the diagonal elements are not equal to 1. As we will discuss in the Section 3, this may lead to a decrease in performance compared to a corresponding KRR model. If the self-similarity terms *k*(**x**_*i*_, **x**_*i*_) and *k*(**x**_*j*_, **x**_*j*_) in eq 6 are high in the extensive kernel, the least dissimilar items based on the kernel-induced distance will be different than the most similar items based on the kernel function, causing the kernel-based *k*‑NN and KRR models to produce different results. This could, in principle, be mitigated via normalizing the kernel such that the diagonal elements are always equal to 1, but this normalization loses the extensiveness property of the kernel and can hence also decrease accuracy.

The other option we considered is metric learning. As the name suggests, the goal of this approach is to learn a distance metric that can distinguish between similar and dissimilar data points in a meaningful way regarding a specific learning goal. In regression, a natural choice is to find a distance metric such that data points with similar labels have short distances between each other and vice versa for data points with dissimilar labels. In this paper, we employ the Metric Learning for Kernel Regression (MLKR) algorithm^52^ as our metric learning algorithm of choice. Originally developed for kernel regression, the goal of MLKR is to learn a Mahalanobis metric that minimizes the leave-one-out quadratic regression error. The objective function to minimize is defined as

$$L=\sum_{i=1}^{n}(y_{i}-f_{\mathrm{LOO‐KR}}(x_{i}){)}^{2}$$ 8

where *f*_LOO‑KR_ is the leave-one-out kernel regression (LOO-KR) model, defined as

$$f_{\mathrm{LOO‐KR}}(x_{i})=\frac{\sum_{j\neq i}y_{j}k(x_{i},x_{j})}{\sum_{j\neq i}k(x_{i},x_{j})}$$ 9

and

$$k(x_{i},x_{j})=\frac{1}{\sigma \sqrt{2\pi }}\exp\left(-\frac{d_{M}(x_{i},x_{j})}{\sigma^{2}}\right)$$ 10

is the standard RBF kernel. The learnable parameter in MLKR is the positive semidefinite matrix **M** that defines the Mahalanobis distance *d*_**M**_:

$$d_{M}(x_{i},x_{j})=(x_{i}-x_{j}{)}^{T}M(x_{i}-x_{j})$$ 11

The Mahalanobis distance is a generalization of the Euclidean distance metric, with an added linear transformation of the input space. For efficiency, **M** can be decomposed into a matrix product (**M** = **A** ^*T*^ **A**) to ensure positive semidefiniteness without expensive checks after each optimization step. Then the distance measure can be trained using standard gradient-based optimization methods with the following explicit gradient:

$$\frac{\partial L}{\partial A}=4A\sum_{i=1}^{n}(f(x_{i})-y_{i})\sum_{j=1}^{n}(f(x_{j})-y_{j})k(x_{i},x_{j})(x_{i}-x_{j})(x_{i}-x_{j}{)}^{T}$$ 12

Both KR and reciprocal distance-weighted *k*-NN models produce predictions in the form of

$$f(x_{i})=\sum_{j=1}^{n}w(d_{M}(x_{i},x_{j}))y_{j}$$ 13

where *w*(*d*_**M**_(**x**_*i*_, **x**_*j*_)) is a weighing function of the distance between test and train instances;

$$w(d_{M}(x_{i},x_{j}))=\frac{d_{M}^{-1}(x_{i},x_{j})}{\sum_{l\in N_{k}(x_{i},d_{M})}d_{M}^{-1}(x_{i},x_{l})}$$ 14

in the case of *k*-NN and

$$w(d_{M}(x_{i},x_{j}))=\frac{k(d_{M}(x_{i},x_{j}))}{\sum_{l=1}^{n}k(d_{M}(x_{i},x_{l}))}$$ 15

in the case of kernel regression. In other terms, with an RBF kernel, the weights of the kernel regression model correspond to a softmax function of the distance. If we make the (admittedly strong) assumption that the kernel similarities of the structures drop off quickly as the distance increases, we can see how the set of nonzero kernel elements overlaps with the set of nearest neighbors for the *k*‑NN model. Furthermore, while the weighing functions are not identical, they are still monotonically decreasing as *d*_**M**_ increases. Hence, we argue that the metric learned by the MLKR metric learning algorithm is also helpful for the *k*‑NN. Indeed, in Section 3, we demonstrate empirically how the *k*-NN model with MLKR learned metric can, in fact, outperform the corresponding kernel regression model.

Learning the MLKR metric introduces an additional computational cost that scales as $O\left(n^{2}p^{2}\right)$ , where *p* is the dimensionality (i.e., the number of features) of the input data. However, as Weinberger et al.^52^ have shown, *p* can be limited via the definition of the matrix **M** and with negligible accuracy penalty. This is conceptually similar to using PCA to reduce the dimensionality of the data. In our case, we found that limiting *p* to 50 did not affect performance; hence, we use this value in all subsequent calculations.

The main hyperparameter in a *k*‑NN model is, naturally, the number of neighbors *k*. Recent work by Kanagawa^53^ has demonstrated how tuning *k* can be achieved with minimal additional computational cost, leveraging the properties of the *k*‑NN formulation. Indeed, calculating the leave-one-out cross-validation score for a data set with a *k*‑NN model is equivalent to scaling the prediction from a (*k* + 1)‑NN model. Hence, if we calculate the full distance matrix between training data points once, an $O\left(n^{2}\right)$ operation, we can then choose an optimal *k* with negligible computation. Alternatively, we can use a separate validation set and choose *k* that provides the smallest validation set loss, which is typically preferable if *n* is large.

Another benefit of *k*‑NN models is that they lend themselves very naturally to uncertainty quantification. As the prediction of such a model is constructed based on taking the mean of the set of nearest neighbors, we can estimate other statistical properties of the same set, such as variance or quantiles, just as easily. Admittedly, if the number of nearest neighbors is small, these estimates will be inaccurate. However, with sufficiently dense and smooth data, higher values of *k* are preferred, and the problem is less pronounced. Alternatively, for uncertainty quantification, more neighbors may be included, and the neighbors can be sampled using procedures such as bootstrap^54^ or jackknife.^55^

#### 2.1.4 Molecular Descriptors

To employ machine learning on the task of predicting chemical properties, we need a way to express the chemical system numerically. Many ways to translate the complex three-dimensional structures and physical properties into such representations or *descriptors* exist. In general, a descriptor is a vector or a tensor which encodes chemical information on the system. Initially, much emphasis in machine learning for chemistry was placed on *global* descriptors,^56^ which encode each system using a set of common features, due to their ease of use with standard machine learning approaches. While global descriptors have been very successful, the so-called local descriptors have received increased attention recently. Instead of encoding the entire chemical system as a whole, local descriptors instead describe the system through encoding individual atoms or groups thereof. Local descriptors are naturally better suited for handling data sets with varying sizes of chemical systems, as they inherently express systems as a series of smaller features. Furthermore, if the target property can be assumed to be decomposable into a sum of atomic contributions, machine learning methods can be adapted to learn these atomic contributions instead of directly inferring the label from the whole system. This has been shown to work well in practice. In particular, kernel-based methods such as KRR can easily be adapted to benefit from this additive property.^48^

In this work, we focus mainly on the FCHL representation^51,57^ (both local and global variants). The FCHL representation has previously been tested and found to perform well with KRR, specifically in predicting the properties of atmospheric clusters.^39^ There exist two formulations of the FCHL representation: the original, later renamed FCHL18 by the publication year, and the more efficient, discretized version, termed FCHL19. In both FCHL representations, the atomic system is expressed as a combination of normal distributions over the first three M‑body interatomic expansions of the system. The first-order expansion encodes the properties of individual atoms, i.e., normal distributions along the rows and columns of the periodic table, whereas the two-body expansion corresponds to the interatomic distances. Finally, the three-body expansion encodes the angular distribution of the system, along with other structural information. The key assumption behind the descriptor is that many properties can be approximated as the sum of these many-body terms. FCHL18 utilizes the first three expansions and produces a three-dimensional tensor as the representation for each system. FCHL19 aims to reduce the size of the representation by discretizing the distributions and omitting the first-order expansion term, with minimal impact on ML predictive accuracy. The result is a set of vectors which encode the atomic environment of each atom in a chemical system. While the FCHL18 representation is always local, summing over the atomic vectors of FCHL19 produces a single, global representation of the system, which can be used in a wide range of machine learning methods.

Several other molecular descriptors—namely the Coulomb Matrix (CM), Bag of Bonds (BoB), and Many-Body Distribution Functionals (MBDF)—are described and employed exclusively in the SI.

### 2.2 Calculation Methodology

#### 2.2.1 Technical Implementation

In this section, we give a brief overview of the technical implementation of the calculations, the results of which we present in the following section.

In our calculations, we experimented with different molecular descriptors and found FCHL19 to perform consistently best across our set of data sets. For a performance comparison between representations, we refer to Section S1 in the Supporting Information. Hence, all ML models use FCHL19 representation as the input data. KRR and kernel-induced metric-based *k*‑NN models use the local (tensor) FCHL19 representation. For implementations requiring a global representation (MLKR and Euclidean distance-based methods), we sum over atomic contributions in the FCHL19 tensor to get a vector representing the chemical system. The ground truth labels are produced via QC simulations, with levels of theory detailed in Section 2.2.2. Each of the QC simulations have been run until convergence to the standard of the respective software implementation.

The computations are conducted using 5-fold cross-validation, as we found this to provide sufficiently robust statistics with a reasonable computational cost. After dividing data into *k*_CV_ = 5 equally sized parts, one chunk is used as hold-out test data. The size of the test set remains fixed in each of the computations. To generate learning curves, the size of training data is varied by subsampling the remaining 80% of the data.

In each of the computations, we use a KRR model with a standard RBF kernel on local representations as a baseline. The two main hyperparameters for KRR, the RBF kernel standard deviation σ and the ridge penalty λ were found via grid search on a training set of 4000 items with a test set of 1000 items. As for the *k*‑NN implementations, we train three distinct models: one using the kernel-induced distance based on the local FCHL19 representation, another model using the MLKR metric learning algorithm of the global FCHL19 representation, and a standard *k*‑NN model on the same global representation and Euclidean distance to find the nearest neighbors. For all the *k*‑NN models, we found that weighting the labels of nearest neighbors by the reciprocal of the distance provided higher accuracy than uniform weights. The number of neighbors for each data set was found using a 5-fold cross-validated hyperparameter search with a random subsample of 5000 items. As MLKR optimizes the metric based on kernel regression loss, we also include a kernel regression model with the MLKR metric in our analysis to study whether the *k*‑NN step adds or detracts from the performance of the model. In all calculations, the MLKR algorithm was run until convergence.

We also tested Δ-learning (discussed in Section 2.1.2) with both KRR and *k*‑NN models. In the following section, whenever Δ-learning is applied, we aim to predict the residual, or difference between labels, of two quantum chemical levels of theory. In our analysis, we chose the levels of theory based performance in previous publications featuring the data sets we use.^39,40^ While the choice of the levels of theory affects the overall error,^49^ in this contribution, we aim to study the relative differences among ML methods. Additionally, the difference among Δ‑learning schemes on the used data sets is smaller than the difference between direct and any form of Δ‑learning. Hence, we have chosen to leave comprehensive comparisons between different Δ‑learning schemes to contributions dedicated to the subject. The levels of theory used with each data set are specified in the corresponding sections.

The calculations were performed on the Grendel cluster (http://www.cscaa.dk/grendel/), a computing cluster maintained by the Centre for Scientific Computing Aarhus at the University of Aarhus, using Intel Xeon CPUs at 2.6–3.0 GHz clock speed.

The methodology is implemented as a part of the JK software framework,^58,59^ and the code to reproduce the calculations presented here can be found at https://github.com/edahelsinki/JK-kNN/.

#### 2.2.2 Data Sets

##### QM9

The QM9 data set^60^ of 134,000 small organic molecules is a widely used, high-quality chemical data set. The data set comprises molecules of H, C, F, N, and O (≤9 atoms), with 15 properties calculated at the B3LYP/6–31G­(2df,p) level of theory. As this data set has been well studied in the literature, we include it as a benchmark for our method. As the target label, we use atomization energy (internal energy at 0 K) directly (i.e., without applying Δ‑learning).

##### Sulfuric Acid–Water Systems

Sulfuric acid (SA) is known to be a main contributor to new particle formation^61^ (NPF), both in continental regions and over oceans. It has a low saturation vapor pressure and a high ability to form molecular clusters with many other molecules, such as various bases. The sulfuric acid–water (SA–W) system is considered the simplest system relevant to understanding the first steps of NPF. Even for such a simple binary system, there are numerous possible configurations of the molecular clusters, which makes KRR methods computationally unfeasible.

We reused the database constructed by Kubečka et al.,^62^ where representative SA–W cluster configurations are evaluated at ωB97X-D/6–31++G­(d,p) and GFN1-xTB levels of theory. The former is a commonly applied and well-benchmarked density functional theory (DFT) method suitable for sizable atmospheric molecular cluster systems.^63–66^ As the basis set is relatively small, the energy is often refined at a higher level of theory, but this method is known to provide accurate equilibrium geometries. The latter is less accurate but a fast semiempirical method (using a tight-binding DFT approach).

We examined ML techniques either by directly predicting the electronic binding energy of the SA–W clusters at the ωB97X-D/6–31++G­(d,p) level of theory or by using a Δ‑learning approach, where the residual is between the aforementioned level of theory and the lower GFN1-xTB level.

##### Clusterome

The main benefit of the increased computational efficiency of a machine learning model is that it allows the model to handle larger data sets. Therefore, the final data set we examine, *Clusterome*, was chosen to demonstrate the scalability of our approach. Clusterome is an atmospheric cluster data set produced using the Clusteromics *I*–*V* data sets^67–71^ and published in a combined form by Knattrup et al.^40^ This data set consists of unique atmospheric acid–base cluster structures, with sulfuric acid (SA), methanesulfonic acid (MSA), nitric acid (NA), and formic acid (FA) as acids and ammonia (AM), methylamine (MA), dimethylamine (DMA), trimethylamine (TMA), and ethylenediamine as bases. The Clusteromics *I*–*V* data sets contain 22,870 equilibrium structures obtained at the ωB97X-D/6–31++G­(d,p) level of theory, and Knattrup et al. expanded this data set to 251,554 entries by including out-of-equilibrium structures obtained from short MD simulations performed at the GFN1-xTB level of theory. In the calculations, we use the Δ‑learning approach with the target being the residual between the ωB97X-D/6–31++G­(d,p) (high) and GFN1-xTB (low) levels of theory.

## 3 Results and Discussion

### 3.1 Learning Atmospheric Molecular Clusters

#### 3.1.1 Accuracy and Computational Cost

First, we compare the performance of different ML models with both direct and Δ‑learning on the data set of sulfuric acid–water (SA–W) clusters. Figure 2 shows the learning curves for the KRR, MLKR, and *k*-NN methods. Here, the mean absolute error (MAE) is calculated with respect to the ground truth QC simulation(s). Δ‑learning leads to a shift in the learning curves with ∼2-times better accuracy compared to direct-learning. However, the overall trends remain the same. Similar to Kubečka et al.,^39^ the KRR model nearly achieves chemical accuracy with *n* = 13,000 training items when using the labels from the high level of theory directly, with mean absolute error decreasing to 0.46 kcal/mol when using Δ‑learning.

**Figure 2:**
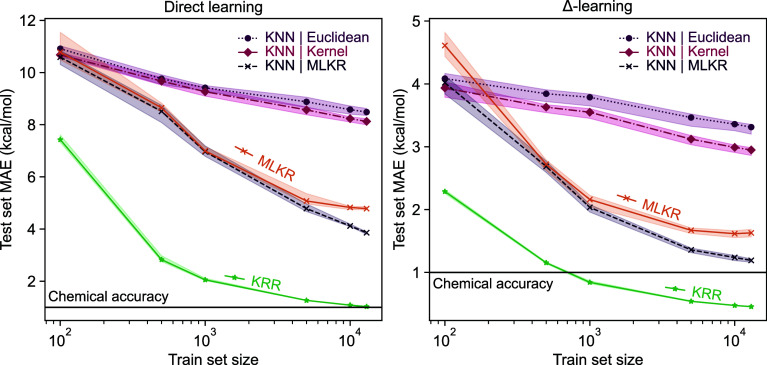
Learning curves or mean absolute error (MAE) with respect to the QC simulation(s) as a function of training set size for direct- and Δ‑learning of electronic binding energies for sulfuric acid–water clusters. The black solid line denotes chemical accuracy (MAE = 1 kcal/mol).

As for the *k*-NN implementations, the approaches based on MLKR metric learning outperform both the kernel-induced distance and standard *k*‑NN variants. In direct learning, the MLKR-based *k*‑NN model reaches a mean absolute error of 3.86 kcal/mol. In Δ-learning, the difference in performance between KRR and *k*‑NN models is less pronounced, with the best *k*‑NN implementation (MLKR with FCHL19) nearly reaching the mark for chemical accuracy of mean absolute error of 1 kcal/mol. Additionally, pairing the *k*‑NN model to the MLKR outperforms the MLKR-based kernel regression model in both direct and Δ‑learning, with the gap growing wider as the size of training data increases. Finally, based on our observations, these models do not exhibit obvious correlations between regression error and chemical properties, namely cluster size or ground truth energy.

While the *k*‑NN models do not reach the same level of accuracy as KRR models, as presented in Figure 3, *k*‑NN may have a significant computational edge on large databases. Naturally, the Euclidean distance-based *k*‑NN far overperforms all other variants in computational costs. However, as predicted by the theory, the training time for KRR and the other *k*‑NN approaches scales differently, with KRR requiring 100 times more CPU time than the slowest *k*-NN implementation already *n* = 5000 training data points. Moreover, as the KRR training in the limit scales as $O\left(n^{3}\right)$ with respect to the data set size, the method quickly becomes entirely computationally intractable. For the learning algorithms paired with MLKR, there is a decrease of 2 orders of magnitude in inference time compared to KRR. More crucially, the inference time also shows similar speed gain for the MLKR metric learning algorithms, with a smaller yet substantial increase for the kernel-induced distance approach as well. Overall, based on the results presented, the MLKR-based *k*‑NN approach combines good predictive performance with impressive computational efficiency.

**Figure 3:**
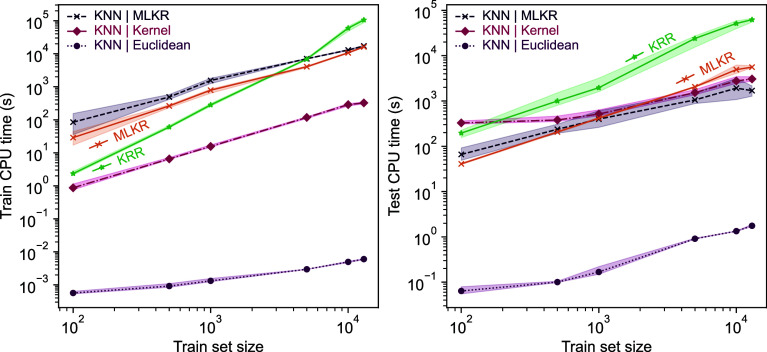
Computation times for direct-learning of electronic binding energies for SA–W clusters. For simplicity, we present computation times only for direct-learning, as direct- and Δ‑learning times are very similar. Notice the logarithmic scale on the *y*-axis.

The kernel-induced distance-based *k*‑NN model does not significantly outperform the Euclidean-distance-based variant. This was unexpected given the strong performance of the KRR model using the kernel as a similarity metric. We offer two hypotheses for the poor performance. First, the KRR model can take into account the entire training data, whereas a *k*‑NN necessarily has a hard cutoff and cannot use information beyond the *k* nearest neighbors. Additionally, the kernel-induced distance may not align with kernel values in unnormalized extensive kernels. We also experimented with normalizing the kernel before calculating the kernel-induced distance, but this did not improve results. Normalizing the kernel did not improve results, suggesting useful information for the *k*‑NN model is lost.

#### 3.1.2 Number of Nearest Neighbors

The choice of how many nearest neighbors to consider (*k*) is pivotal for the model’s performance. A small *k* represents a more flexible model, but is more prone to overfitting; a high value for *k* results in more robust but less expressive models. In the learning curve calculations above, the value of *k* = 12 was chosen based on a hyperparameter search described in Section 2. To ensure that the optimal *k* does not depend on the size of the training data set, we reran the calculations for different values of *k*. Figure 4 shows how the mean absolute error of various *k*‑NN implementations rapidly decreases as *k* increases but quickly plateaus for the MLKR-based *k*‑NN model. Furthermore, the optimal *k* value remains practically constant across different data set sizes. This suggests that, especially for large data sets, the model performance is not sensitive to the choice of *k*, provided that it is reasonable (e.g., 5 ≤ *k* ≤ 15). Hence, for users, if hyperparameter optimization (e.g., using a similar procedure as described here) is infeasible, we recommend setting *k* = 10 as the first guess when using an MLKR-based *k*‑NN model.

**Figure 4:**
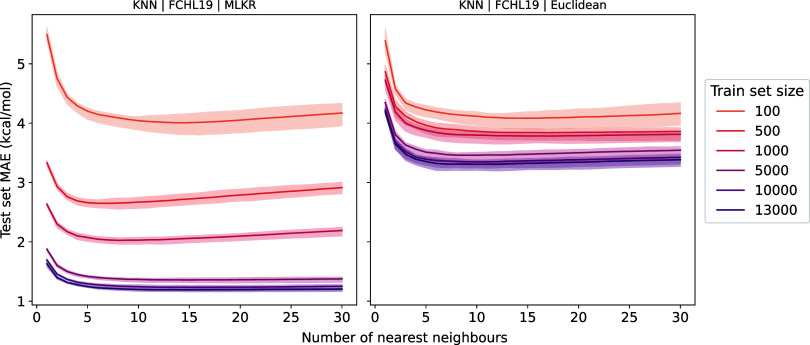
Test mean absolute error as a function of *k* at different training set sizes (colored lines) on the SA–W Δ‑learning data for both the MLKR-based *k*‑NN and the Euclidean variant. The choice of *k* has little impact, especially as the data set size grows, provided it is reasonably chosen (i.e., 5 ≤ *k* ≤ 15).

The Euclidean variant is more sensitive to the choice of *k*, even at 13,000 items. Nonetheless, the optimal value of *k* remains constant even for this variant.

#### 3.1.3 Modeling Large Clusters

To compare the extrapolation performance of various *k*‑NN implementations against KRR, we trained models on the SA–W data with Δ‑learning similar to Section 3.1.1, excluding the largest (SA)_4_(W)_5_ clusters, and then attempted to predict the electronic energies of these holdout data points. As Figure 5 shows, the difference between KRR and *k*‑NN is more pronounced in extrapolation compared to interpolation (cf. Figure 2); the KRR model reaches a mean absolute error of 0.63 kcal/mol at 13,000 training items while the best-performing MLKR-based *k*‑NN model has an error of 1.63 kcal/mol at the same level. This decrease in accuracy can be explained primarily via two different effects. First, as shown in the previous section and in Figure 2, the *k*‑NN approaches, while more computationally scalable, are more data-intensive than KRR. Hence, for similar *k*‑NN and KRR performance, we expect *k*‑NN to require more data. Second, there may be a fundamental difference in the extrapolation capability between the ML models. However, as the slopes of the learning curves between the KRR and MLKR-based *k*‑NN model remain similar, such fundamental differences seem to have only a limited effect. In conclusion, while the performance of the *k*‑NN model drops on the more difficult extrapolation task, the decrease is relative to that of the KRR model and the larger error for the *k*‑NN can be explained for the most part by the fact that the method requires more data to achieve similar performance.

**Figure 5:**
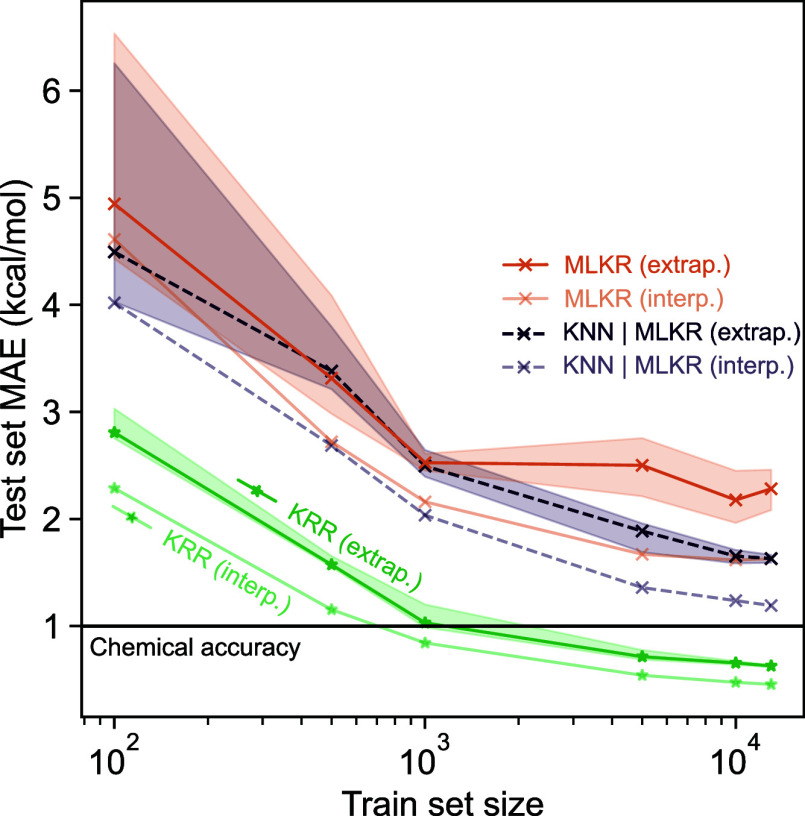
Extrapolation—learning curves for the SA–W cluster, where instead of using cross-validation (cf. Figure 2), the largest (SA)_4_(W)_5_ clusters are used for test, while the remaining smaller clusters are used for training. The faint lines show the learning curves for Δ‑learning on the cross-validated data (interpolation task).

#### 3.1.4 Interpretability and Uncertainty Estimation

The nearest neighbor sets of a *k*‑NN model can be analyzed for model interpretation and uncertainty estimation. From these sets, for example, we may estimate the deciles as a representation of the prediction uncertainty. Furthermore, the set of nearest neighbors can be manually inspected to learn which items the model deems most similar and to ensure that this neighbor set aligns with domain expectations.

To demonstrate these qualities, we study a cross-validation fold from the Δ‑learning calculation using the SA–W data set, trained on 13,000 structures and tested on 3471 previously unseen structures. The *k*‑NN model in question is MLKR-based, and we used the FCHL representation. In the upper left corner of Figure 6, we show a scatter plot of the true label value of items in the test set vs the residual (error) between the true value and the prediction. Ten items show the 50% confidence intervals, i.e., the range between the 25th and 75th percentiles of the labels in the neighbor set of these ten items, as error bars, with a red dashed line indicating zero error. For most items, the true label is either included in or close to the error bars, implying that the confidence intervals derived from nearest neighbors are meaningful.

**Figure 6:**
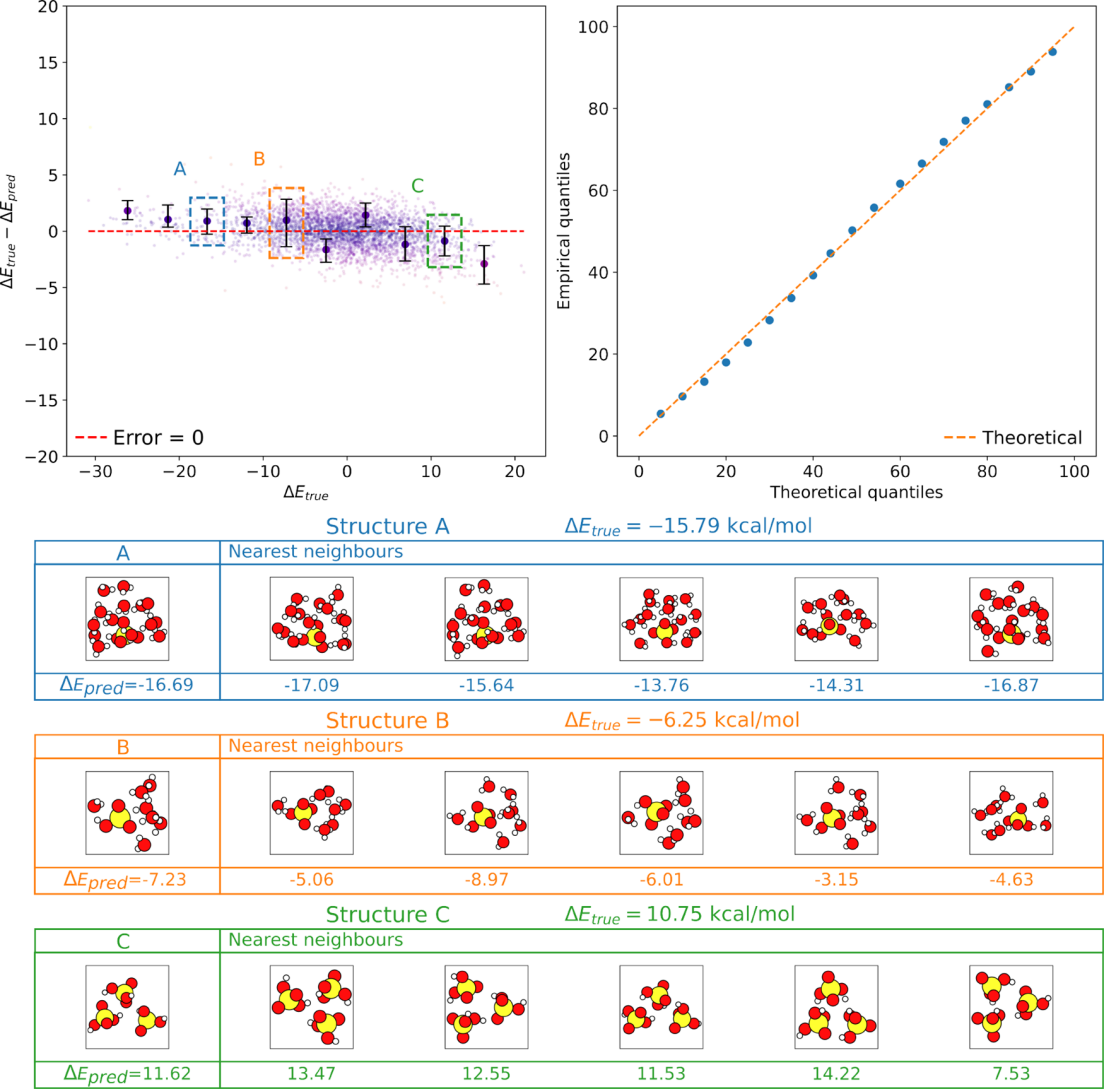
Demonstration of uncertainty estimation and interpretations of a *k*‑NN model. The upper left panel shows the true label value vs absolute error of an MLKR-based *k*‑NN model trained on the SA–W data, with ten items additionally showing error bars based on 50% confidence intervals from the set of nearest neighbors for each item and a red line indicating zero error. The upper right panel shows a calibration plot for the quantiles, where blue dots show the percentage of test labels falling below each empirical percentile. Finally, the bottom half shows the structures along with the true and predicted label values for three example items in the test set (indicated by colored boxes).

To further ensure that the quantiles for the target value from the neighbor sets are properly calibrated, the upper right corner in the same figure shows a calibration plot for the quantiles. In this context, calibrated means that our quantiles correspond to the statistical properties of the data. For each item, we first estimate quantiles based on the set of unweighted nearest neighbors in the training data set. We then compare these quantiles to the true labels on the test set, which are previously unseen by the model. If the true label is below the estimated fifth percentile approximately in one in 20 items (i.e., 5%) in the test set and so on for other percentiles, the quantiles can be considered well calibrated. As the upper right panel shows, the proportion of items in the test below the corresponding percentile value (blue dots) follow the theoretical percentiles (red dashed line) closely, demonstrating good calibration.

Finally, the bottom half of Figure 6 shows the five nearest neighbors for three example structures, indicated by colored boxes in the upper left panel. As can be seen, the model has learned to find structures of similar composition acting as a sanity check for the model performance. Conversely, clear outliers could be easily identified as items with inconsistent neighbor sets and large distances to their nearest neighbors. We will not delve further into analyzing the chemical properties of these particular structures as that is not the main aim of the paper; we seek to demonstrate how the *k*-NN modeling approach allows for interpretation of results by examination of the nearest neighbor set by the user.

### 3.2 Learning Large Data Sets

The Clusterome data set is both a larger and more complex version of the SA–W cluster data discussed in the previous section. Figure 7 presents the learning curve and computational cost for the tested models on the Clusterome data set. Expanding the training set size beyond 100,000 clusters increases the computational cost, which should give the *k*‑NN models an advantage. Furthermore, we found that capping the MLKR metric learning to a random subsample of 25,000 items introduces only a negligible cost to accuracy; the *k*‑NN model then uses the learned metric with all available training data. For example, at 50,000 training items, using the full training set to learn the MLKR metric resulted in a MAE of 1.30 kcal/mol in contrast to 1.36 kcal/mol for the subsampled variant; only a 4.6% decrease. Performing subsampling does, however, cut the training time drastically; as expected, for the set 50,000 items, the subsampled variant required, on average, only 26% of the time needed to train the nonsubsampled variant.

**Figure 7:**
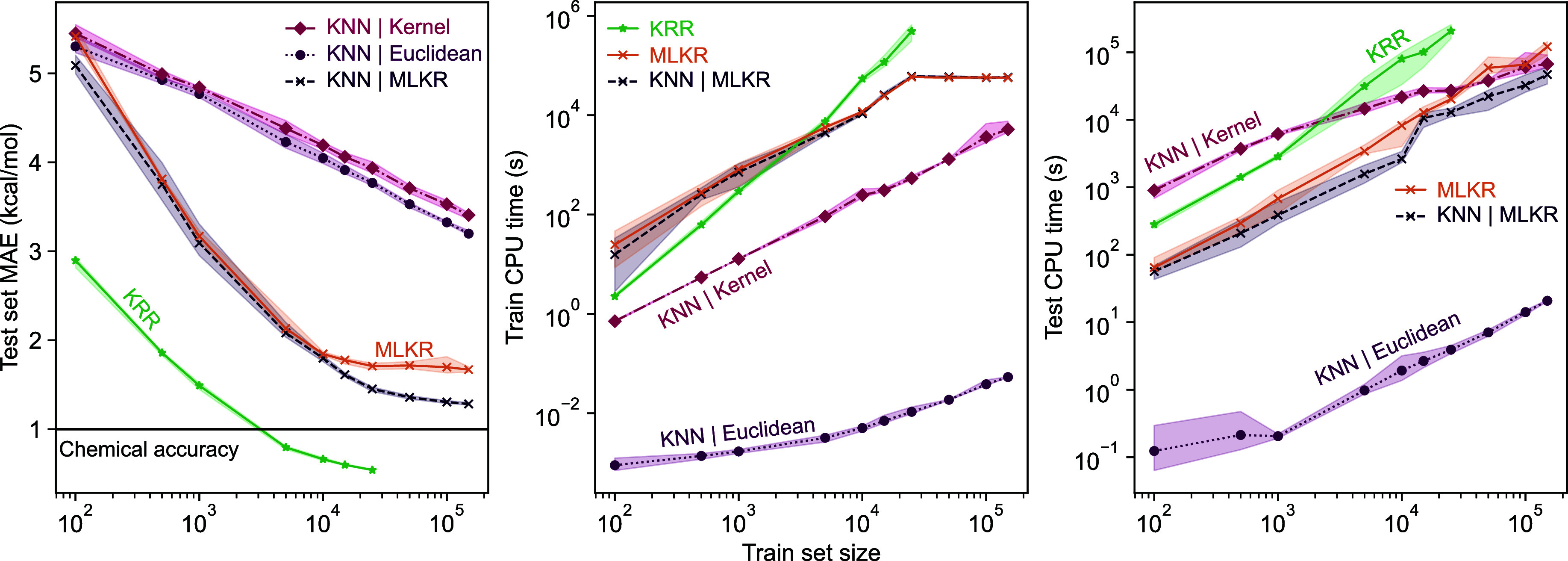
Learning curves and computational cost for Δ‑learning the electronic binding energies of the Clusterome data set. The ground truth labels are produced with QC simulations, with levels of theory detailed in Section 2.2.2. The computational advantage of *k*‑NN approaches can be seen clearly. In MLKR and MLKR-based *k*‑NN, we have limited the metric learning to a subsample of 25,000 items.

The larger training set size illustrates the difference in scaling computational cost. As the size of the training set grows, the difference in training time between MLKR-based *k*‑NN models and KRR widens by orders of magnitude, even before considering the subsampling for MLKR metric learning. Accuracy-wise, while the KRR model outperforms the *k*‑NN approaches here, as well, we can still nearly reach chemical accuracy with the MLKR-based *k*‑NN model while handling many times more data than would be computationally feasible for KRR.

### 3.3 Learning the QM9 Compound Data Set

The learning curves for all models trained on the QM9 data set are shown in Figure 8. As mentioned in Section 2.2.2, the test set size is fixed to 26,777 items; the training set size varies. The KRR model shows similar performance to Christensen et al.,^57^ who introduced the FCHL19 representation, ensuring that our implementation is correct. The KRR model reaches the level of MAE ≈ 1 kcal/mol with around 10,000 training samples. The best-performing *k*‑NN variant, using MLKR metric learning, reaches an MAE of 3 kcal/mol only at 25,000 samples, albeit with much lower computational cost. Training for both MLKR-based *k*‑NN model and the KRR model at 25,000 training samples took 60,000 CPU-seconds, on average, and predicting the test set of 26,775 points took 57,000 CPU-seconds for the *k*‑NN and 162,000 CPU-seconds for the KRR; nearly triple the computational cost.

**Figure 8:**
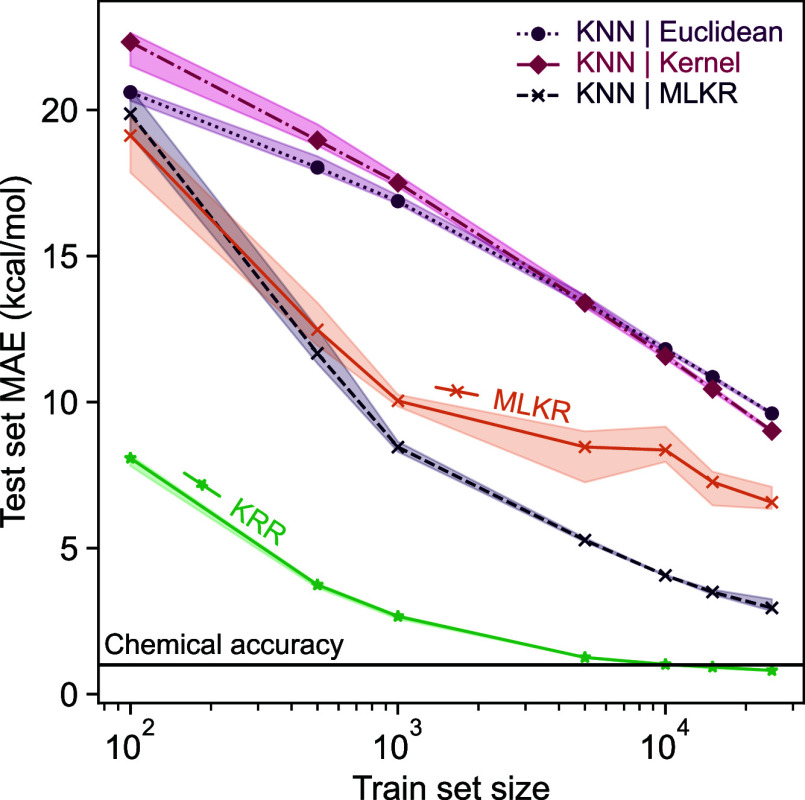
Learning curves for the atomization energies of the QM9 data set with respect to the QC simulation. The shaded area reflects the error variance.

As expected, the Euclidean-distance-based *k*‑NN model performs far worse than the metric-learning variant, with a top mean absolute error of 9.6 kcal/mol, albeit with minimal computational cost; training the model at 25,000 training items took less than 0.01 CPU-seconds and predicting the test set less than 20 CPU-seconds, on average. The kernel-induced distance-based *k*-NN model shows similar performance to the Euclidean-distance-based variant.

Finally, as before, the results show how the MLKR-based kernel regression model does not reach the accuracy of the MLKR-equipped *k*‑NN model, demonstrating that combining the models yields gains in accuracy. As most of the computational budget is spent on learning the distance metric, the computational cost of MLKR-kernel regression is very similar to the *k*‑NN variant both in training and prediction.

## 4 Conclusions

Finding novel, efficient, and interpretable ways to predict the properties of large chemical systems would unlock greater understanding of the complex processes affecting the climate and our health. In this section, we have demonstrated how *k*‑NN regression, a classic yet often overlooked machine learning approach, offers a highly efficient and more interpretable alternative to the widely used KRR models as a tool for machine learning in chemistry. By integrating chemically informed distance metrics, in particular MLKR-based metric learning, we show that *k*‑NN models can achieve near-chemical accuracy while dramatically reducing computational overhead. In particular, our approach scales effortlessly to data sets exceeding 250,000 entries and performs robustly even in extrapolation tasks involving large molecular clusters. This enhanced computational efficiency, combined with model transparency, makes such models an enticing alternative, especially in data-rich, interpolation-oriented settings.

As curated data sets continue to grow, we anticipate that hybrid workflows leveraging fast, interpretable instance-based models like *k*‑NN will become an increasingly valuable component of molecular computational chemistry pipelines with further applications, e.g., in atmospheric chemistry. Future work could look into ways to encode prior information (such as cluster composition) more explicitly into the metric learning layer or coupling it with scalable approximate nearest-neighbor methods for ultralarge-scale applications. Notably, applying *k*‑NN-based models to molecular dynamics (MD) simulations, by learning not only energies but also forces, would be a promising avenue. This would allow for transparent and computationally efficient MD models without relying on less interpretable architectures, such as neural networks, and settings where KRR becomes computationally intractable.

## Supplementary Material



## Data Availability

All the code necessary to recreate the calculations is available at https://github.com/edahelsinki/JK-kNN/

## Notes

Section S1: comparison between the performance of molecular representations other than FCHL19 (PDF)
