# Supplementary material for: Fast and Interpretable Machine Learning Modeling of Atmospheric Molecular Clusters
Source: J Phys Chem A. 2026 Jan 15;130(4):902–13. doi: 10.1021/acs.jpca.5c06950 (PMC12862803; doi:10.1021/acs.jpca.5c06950)
Supplement: Supplementary file 1 [file jp5c06950_si_001.pdf]

Supporting Information: Fast and Interpretable Machine Learning Modelling of Atmospheric Molecular Clusters

Lauri Seppäläinen,[*,†] Jakub Kubečka,[‡] Jonas Elm,[‡] and Kai R. Puolamäki[†]

†Department of Computer Science, Pietari Kalmin katu 5, University of Helsinki, Helsinki, FI 00560

‡Department of Chemistry, Aarhus University, Langelandsgade 140, Aarhus C, DK 8000

E-mail: lauri.seppalainen@helsinki.fi

S1 Molecular representation comparison

The choice of representation is a crucial step in applying ML on chemical data. While the FCHL representation was introduced in the main body of the text, we also included the Coulomb matrix (CM), Bag-of-Bonds (BoB) and many-body functional distributions (MBDF) representations in our analysis for completeness.

Coulomb matrices[1] are a simple yet effective molecular representation. The representation is global and aims to mimic the electrostatic interaction between nuclei in a chemical system. Bag-of-bonds[2] takes inspiration from the bag-of-words representations common in natural language processing in computer science where the electrostatic interactions between nuclei are bagged to produce a single vector as the representation. Similar to FCHL19, the MBDF[3] representation is designed to minimise the size of the representation without dramatically impacting predictive accuracy. Furthermore, both representations aim to capture the distribution of the many-body properties of the chemical system. While FCHL encodes each of these features explicitly, MBDF instead expresses the system through a finite set of integrals that describe these distributions. Conceptually, FCHL provides a high-dimensional, explicit encoding of the local atomic environments by modelling the distributions of elements, distances, and angles. In contrast, MBDF summarizes these distributions by computing statistical moments (e.g., mean, variance) to produce a lower-dimensional representation.

Table S1 shows the test set mean absolute error and train and test CPU times of k-NN models trained on $13,000$ samples of Δ-learning data from SA–W clusters (identical to Section 4.2.1 in the main paper) and with various representations and metric types. As we have alluded in previous sections, FCHL19 combined with the MLKR metric learning offers good accuracy with reasonable training time. MBDF, on the other hand, does not perform as well and produces results similar to the much simpler bag-of-bonds.

While plain FCHL18 is unsuitable to be used with Euclidean or MLKR-based k-NN

models due the representation being a 4D tensor, we did experiment with kernel-induced distance using FCHL18 as well. As the table shows, the FCHL18-based kernel-induced distance k-NN achieves similar results to FCHL19 (i.e., nearly within one standard deviation), but is much slower due to the more complicated calculation of the kernel elements in both training and testing.

Table S1: Comparison of representations and metric types in k-NN model applied to the SA–W dataset. Smaller values are better in each column, with best values highlighted in bold. The representations include bag-of-bonds (BoB), Coulomb matrices (CM), both FCHL18 and FCHL19 variants and MBDF. The metric types include Euclidean distance, MLKR-based learned metric and for FCHL18 and FCHL19, kernel-induced distance (denoted "Kernel" in the table).

Representation	Metric type	Test MAE (kcal/mol)	Train CPU time (s)	Test CPU time (s)
BoB	Euclidean	3.28 ± 0.05	0.01 ± 0.00	3.04 ± 0.04
	MLKR	2.25 ± 0.04	10029.99 ± 759.65	2302.37 ± 789.87
CM	Euclidean	3.78 ± 0.07	0.01 ± 0.00	2.61 ± 0.01
	MLKR	3.81 ± 0.07	13253.95 ± 1279.53	2001.73 ± 292.70
FCHL18	Kernel	2.89 ± 0.05	9591.09 ± 215.50	65901.09 ± 625.11
FCHL19	Euclidean	3.31 ± 0.06	0.01 ± 0.00	**1.46 ± 0.01**
	Kernel	2.95 ± 0.05	361.99 ± 47.88	13914.71 ± 1202.50
	MLKR	**1.19 ± 0.02**	14392.87 ± 1855.89	1908.42 ± 331.91
MBDF	Euclidean	2.92 ± 0.08	**0.00 ± 0.00**	1.51 ± 0.09
	MLKR	2.23 ± 0.03	13640.22 ± 277.35	1658.75 ± 225.30

References

(1) Rupp, M.; Tkatchenko, A.; Müller, K.-R.; von Lilienfeld, O. A. Fast and Accurate Modeling of Molecular Atomization Energies with Machine Learning. *Phys. Rev. Lett.* **2012**, *108*, 058301.

(2) Hansen, K.; Biegler, F.; Ramakrishnan, R.; Pronobis, W.; von Lilienfeld, O. A.; Müller, K.-R.; Tkatchenko, A. Machine Learning Predictions of Molecular Properties: Accurate Many-Body Potentials and Nonlocality in Chemical Space. *6*, 2326–2331.

(3) Khan, D.; Heinen, S.; Anatole von Lilienfeld, O. Kernel Based Quantum Machine Learning at Record Rate: Many-body Distribution Functionals as Compact Representations. *159*, 034106.
